# Towards smart energy systems: application of kernel machine regression for medium term electricity load forecasting

**DOI:** 10.1186/s40064-016-1665-z

**Published:** 2016-01-20

**Authors:** Miltiadis Alamaniotis, Dimitrios Bargiotas, Lefteri H. Tsoukalas

**Affiliations:** Applied Intelligent Systems Laboratory, School of Nuclear Engineering, Purdue University, 400 Central Dr., West Lafayette, IN 47907 USA; Department of Electrical Engineering, Technological Institute of Stereas Elladas, 34400 Dimos Dirfion-Messapion, Psachna, Evia Greece

**Keywords:** Relevance vector regression, Gaussian process regression, Medium term load forecasting, Smart energy systems

## Abstract

Integration of energy systems with information technologies has facilitated the realization of smart energy systems that utilize information to optimize system operation. To that end, crucial in optimizing energy system operation is the accurate, ahead-of-time forecasting of load demand. In particular, load forecasting allows planning of system expansion, and decision making for enhancing system safety and reliability. In this paper, the application of two types of kernel machines for medium term load forecasting (MTLF) is presented and their performance is recorded based on a set of historical electricity load demand data. The two kernel machine models and more specifically Gaussian process regression (GPR) and relevance vector regression (RVR) are utilized for making predictions over future load demand. Both models, i.e., GPR and RVR, are equipped with a Gaussian kernel and are tested on daily predictions for a 30-day-ahead horizon taken from the New England Area. Furthermore, their performance is compared to the ARMA(2,2) model with respect to mean average percentage error and squared correlation coefficient. Results demonstrate the superiority of RVR over the other forecasting models in performing MTLF.

## Introduction

Limitations in current power infrastructure together with world-wide concerns, like climate change and economic stability are the driving factors to ongoing research efforts for developing a new generation of smart energy systems (Fainti et al. 2014). Realization of smart energy systems is greatly accommodated by coupling information technologies with power systems. In particular, the advent of internet and advancements in communication technologies inspired the notion of an Energy Internet (Alamaniotis et al. 2011a, b), in which information networks interact with power generation, transmission, and distribution systems aiming at optimizing power system operation.

Smart energy systems utilize information to overcome the significant constraints of the current power grid infrastructure (Tsoukalas and Gao 2008). The limited delivery capacity and the lack of large scale energy storage may lead to grid destabilization causing distribution failures with high financial impact to grid participants. For instance, (i) load demand beyond delivery capacity results in financially expensive system failures and blackouts (Alamaniotis et al. 2014b), and (ii) the amount of excess generated energy that cannot be stored is wasted since the generation does not closely follow the demand (Gao et al. 2003).

Electricity load forecasting has been recognized as a key issue in implementing smart energy systems (Alamaniotis et al. 2014a, b). Load forecasting may be used by all smart grid participants aiming at reaching their goals. For example, consumers utilize load forecasting for consumption planning and scheduling while grid operators for safe and secure electricity delivery. Depending on the forecasting time horizon, load forecasting may be identified as very short term (VSTLF) ranging from some minutes to an hour (Alamaniotis et al. 2012), short term (STLF) (Alamaniotis et al. 2011a, b) ranging from an hour to a week, medium term (MTLF) ranging from a week to a year (Ghiassi et al. 2006), and long term load forecasting (LTLF) for longer than a year ahead of time predictions (Kandil et al. 2002).

The current manuscript focuses on medium term load forecasting. MTLF is an efficient tool for implementing smart energy systems since it promotes optimal expansion planning by considering climate changes, maintenance scheduling, fuel purchase negotiating (for instance for nuclear power plants), component replacing or repairing, and maximizing utilization of renewable resources such as wind power. Furthermore, it is expected to play a crucial role in developing price directed energy markets in which entities will participate via intelligent meters (Gatsis and Giannakis 2012) and require forecasting tools to develop their electricity purchase strategies.

Though the number of proposed approaches for performing MTLF is limited, there are ongoing efforts for developing more sophisticated and advanced tools that satisfy the demands imposed by the advent of the “big data” era. The proposed approaches make use of tools coming from statistics and artificial intelligence fields. A dynamic artificial neural network is proposed in (Ghiassi et al. 2006), and a radial basis function neural network in (Xia et al. 2010), while combination of neural networks with expert systems in (Kim et al. 1995). Other methods employed adaptive neural networks (Tsekouras et al. 2006), particle swarm optimization (Rengcun et al. 2008), and singular value decomposition (Abu-Shikhah and Elkarmi 2011). Nonlinear multivariable regression for MTLF is presented in (Tsekouras et al. 2007), while a combination of linear and non-linear regression for MTLF is introduced in (Abu-Shikhah et al. 2011), and Gaussian processes for a year ahead monthly load forecasting in (Alamaniotis et al. 2014a). Furthermore, a support vector machine based approach for MTLF is discussed in (Bozic and Stojanovic 2011), while a hybrid methodology comprised of autoregressive integrated moving average (ARIMA) and artificial neural network is introduced and tested in (El Desouky and Elkateb 2000). The above methodologies, though effective, come at a cost of high prediction uncertainty. In addition they lack the necessary flexibility to update their predictions since they are unable to capture nonlinear load dynamics.

In this paper intelligent regression models for MTLF are examined. The proposed models make use of machine learning tools and more specifically of kernel machines (Scholkopf and Smola 2001). In particular, relevance vector regression (Tipping 2001) and Gaussian process regression (Rasmussen 2006) are utilized for making predictions for longer than a week ahead of time horizon. Generally speaking, kernel machines are nonlinear methods that inherently make use of semi-positive definite matrices in order to make predictions (Hoffman et al. 2008). They are able of detecting the kind of dependencies that dominate the load properties by formulating the feature space in terms of kernels. Formulation of feature space by kernels is the advantage of kernel machines as opposed to the rest load forecasting methods mentioned earlier; it allows the modeler to control the forecasting process by selecting the kernel form, and promotes model flexibility by offering a high variety of kernels (Alamaniotis et al. 2015). For instance, kernel regression facilitates selection of a kernel that models particular data properties, for example stationarity, in contrast to artificial neural networks that require not only selection of neuron activation functions but also network architecture (Tsoukalas and Uhrig 1997). Assessment of the forecasting performance is done using the mean average percentage error (MAPE) and squared correlation coefficient (*R*^2^), while the testing datasets are comprised of the daily demand for a 30-day-ahead horizon.

The roadmap of the paper is as follows: in the next two sections a brief presentation on kernel machines is provided and the proposed methodology is presented. Medium term load forecasting results are given in the “Results” section, while the last section concludes and summarizes the main points of the paper.

## Background

### Kernel machines

Analytical models that can be expressed as a function of a kernel are known as kernel machines (Bishop 2006). A kernel is any valid mathematical function that can be written with respect to the dual representation. The general form of the dual representation is given by:

1 $$k(x_{1} ,x_{2} ) = \varphi (x_{1} )^{T} \varphi (x_{2} )$$

with $\varphi$(*x*) being any analytical function known as *basis function*, and *k*(*x*, *x*) representing a kernel function. In general, formulating a function using Eq. (1) is known as the kernel trick. A few examples of common kernel functions are the linear and the polynomial kernels whose analytical formulas are given respectively by (Bishop 2006):

2 $$k(x_{1} ,x_{2} ) = x_{1}^{T} x_{2}$$

3 $$k(x_{1} ,x_{2} ) = \left( {x_{1}^{T} x_{2} } \right)^{2} .$$

Beyond the widely known kernels, new valid kernels may be created by composition of two, or more, valid kernels by applying the operations of addition and/or multiplication (Rasmussen 2006). The selection of an appropriate kernel function is a main design choice that must generally be made by the designer according to the specifications of the problem at hand.

### Gaussian process regression

The set of random variables that has a joint Gaussian distribution is defined as a Gaussian process. A Gaussian process is fully determined by its mean m(*x*) and covariance function C(*x*, *x*′), and therefore, the Gaussian process takes the form:

4 $$GP(m(x),C(x,x^{{\prime }} ))$$

where it is common to assume for convenience that m(*x*) = 0.

Gaussian processes are applied in regression problems where they deal with problems of predicting continuous parameters. Derivation of Gaussian process regression (GPR) has as a point of start the simple linear regression:

5 $$y({\mathbf{x}},{\mathbf{w}}) = \sum\limits_{i = 1}^{N} {w_{i} \phi_{i} }$$

where *w*_*i*_ are the regression weights and *φ*_*i*_ are the basis functions. Equation (5) may be written in vector form as given below:

6 $$y_{n} = {\mathbf{\Phi w}}.$$

Next, a prior normal distribution over the model weights is adopted:

7 $$P({\mathbf{w}}) = N({\mathbf{0}},\sigma_{w}^{2} {\mathbf{I}})$$

where **0** represents the mean vector, $\sigma_{w}^{2}$ is the variance equal for all individual weights, and **I** is the identity matrix. Therefore, the distribution over the vector output **y** is also normal:

8 $$P({\mathbf{y}}) = N({\mathbf{0}},{\mathbf{C}}_{y} ) .$$

Regression problems require taking into account noisy observed target values. If *ε*_*n*_ denotes the additive noise with zero mean and variance $\sigma_{n}^{2}$, then the target values become:

9 $$t_{n} = y({\mathbf{x}}^{(n)} ) + \varepsilon_{n} .$$

Hence, the distribution over the target variables is also normal

10 $$P({\mathbf{t}}) = N({\mathbf{0}},{\mathbf{C}}) = N({\mathbf{0}},{\mathbf{C}}_{y} + \sigma_{n}^{2} {\mathbf{I}}).$$

In Gaussian process regression the Bayesian formalism is applied in order to infer a predictive distribution, i.e. a mean value and the associated variance. The prediction over the target *t*_N+1_ for an unknown input **x**^(N+1)^ is based on the previous observed targets **t**_N_ and the respective inputs **x**_N_ and thus the predictive distribution becomes

11 $$P(t_{N + 1} |{\mathbf{t}}_{N} ) \propto \exp \left\{ { - \frac{1}{2}\left[ {\begin{array}{*{20}c} {{\mathbf{t}}_{N} } & {t_{N + 1} } \\ \end{array} } \right]{\mathbf{C}}_{N + 1}^{ - 1} \left[ {\begin{array}{*{20}c} {{\mathbf{t}}_{N}^{T} } \\ {t_{N + 1} } \\ \end{array} } \right]} \right\}$$

where it is apparent that the predictive distribution depends on the inverse of the covariance matrix **C**_N+1_. In order to ease computation of the predictive distribution parameters, the covariance matrix **C**_N+1_ is subdivided into four submatrices (Williams 2002)

12 $${\mathbf{C}}_{N + 1} = \left[ {\begin{array}{*{20}c} {\left[ {{\mathbf{C}}_{N} } \right]} & {\left[ {\mathbf{k}} \right]} \\ {\left[ {{\mathbf{k}}^{T} } \right]} & {\left[ k \right]} \\ \end{array} } \right]$$

with **C**_N_ being the covariance matrix of the *N* observations, **k** being a vector of length *N* encompassing the covariances between the *N* + 1 and each of the rest *N* points, and *k* being the scalar value of the variance of the point *N* + 1. Thus, it can be shown (Mackay 1998) that the parameters of the normal predictive distribution, i.e. the mean and the covariance over *N* + 1, are given by the following formulas respectively:

13 $$m({\mathbf{x}}^{(N + 1)} ) = {\mathbf{k}}^{T} {\mathbf{C}}_{N}^{ - 1} {\mathbf{t}}_{N}$$

14 $$\sigma^{2} ({\mathbf{x}}^{(N + 1)} ) = k - {\mathbf{k}}^{T} {\mathbf{C}}_{N}^{ - 1} {\mathbf{k}}$$

where it is noted that both equations depend on covariance matrix **C**_N_ instead of **C**_N+1_.

### Relevance vector regression

In the current manuscript we consider the regression form of relevance vector machines, which is known as relevance vector regression (RVR). In deriving RVR, initially, we assume that the target variable *t* given an input **x** follows a normal distribution:

15 $$p\left(t|{\mathbf{x}},{\mathbf{w}},\frac{1}{{\mathop \sigma \nolimits^{2} }}\right) = N(t|y({\mathbf{x}}),\mathop \sigma \nolimits^{2} )$$

where σ^2^ is the variance of the data noise while the mean value *y*(**x**) is given by:

16 $$y({\mathbf{x}}) = \sum\limits_{n = 1}^{M} {w_{n} \phi_{n} ({\mathbf{x}})} = {\mathbf{w}}^{T} \phi ({\mathbf{x}})$$

where $\phi ()$ is a valid function called the *basis function*, *M* is the population of basis functions and **w** is the weight vector. By using Eq. (16) and kernel functions, RVR is modeled as below:

17 $$y({\mathbf{x}}) = \sum\limits_{n = 1}^{N} {w_{n} k({\mathbf{x}},{\mathbf{x}}_{n} ) + b}$$

with *b* is the bias term and *N* is the population number of known observations (i.e., size of training dataset). Next, we consolidate the *N* input observations into a single matrix **X**, and the respective *N* outputs into a vector **t**. Thus, we get a likelihood function:

18 $$p\left({\mathbf{t}}|{\mathbf{X}},{\mathbf{w}},\frac{1}{{\sigma^{2} }}\right) = \prod\limits_{n = 1}^{N} p \left(t_{n} |{\mathbf{x}}_{n} ,{\mathbf{w}},\sigma^{2} \right)$$

and a prior distribution over the weight vector **w**:

19 $$p({\mathbf{w}}|{\varvec{\upalpha}}) = \prod\limits_{n = 1}^{M} N (w_{n} |0,\alpha_{n} )$$

with *α*_n_ being the variance of weight *w*_n_ and *M* equal to *N* + 1. At this point we plug into the Bayes formula both Eq. (18) and (19) and hence we get the posterior distribution over **w**:

20 $$p({\mathbf{w}}|{\mathbf{t}},{\mathbf{X}},{\varvec{\upalpha}},\sigma^{2} ) = N({\mathbf{w}}|{\mathbf{m}},{\varvec{\Sigma}})$$

where mean is taken by:

21 $${\mathbf{m}} = \frac{1}{{\sigma^{2} }}{\mathbf{\Sigma \Phi }}^{T} {\mathbf{t}}$$

and respective variance by

22 $${\varvec{\Sigma}} = \left({\mathbf{A}} + \frac{1}{{\sigma^{2} }}{\varvec{\Phi}}^{T} {\varvec{\Phi}}\right)^{ - 1}$$

with **A** = diag(α_i_) and **Φ** = **Κ**; **K** is a (*N* + 1)x(*N* + 1) dimensional matrix with elements given by the kernel function *k*(x_n_, x_m_).

At this point it should be said that the unknown parameters *α*_i_ and *σ*^2^ are evaluated by maximizing the logarithmic marginal likelihood:

23 $$\ln p\left( {{\mathbf{t}}|{\mathbf{X}},{\varvec{\upalpha}},\frac{1}{{\sigma^{2} }}} \right) = \ln N({\mathbf{t}}|0,{\mathbf{C}}) = - \frac{1}{2}\left\{ {N\ln 2\pi + \ln |{\mathbf{C}}| + {\mathbf{t}}^{T} {\mathbf{C}}^{ - 1} {\mathbf{t}}} \right\}$$

where **t** = (*t*_1_,…,*t*_N_)^T^ and **C** is a *N* × *N* dimension matrix given by:

24 $${\mathbf{C}} = \frac{1}{{\sigma^{2} }}{\mathbf{I}} + {\mathbf{\Phi A}}^{ - 1} {\varvec{\Phi}}$$

where **I** is respectively the identity matrix.

Maximization of the marginal likelihood in Eq. (23) with an appropriate iterative method allows evaluation of its parameters. Therefore, the computed optimal values for **α** and σ^2^ are equal to α* and (σ^2^)* respectively. Some of the elements of the vector α* are driven to infinity and thus the posterior distribution of their weights is normal with both mean and variance being equal to zero. As a result, the corresponding kernel functions have no contribution in prediction making driving the output to depend exclusively on the *non*-*zero weighted kernels*. The inputs associated with non-zero weighted kernels are called *relevance vectors*.

Therefore, RVR provides a predictive distribution over the target value *t* of a new input **x**:

25 $$p\left(t|{\mathbf{x}},{\mathbf{X}},{\mathbf{t}},a^{*} ,\frac{1}{{(\sigma^{2} )^{*} }}\right) = N\left(t|{\mathbf{m}}^{T} \phi ({\mathbf{x}}),\sigma^{2} ({\mathbf{x}})\right)$$

with mean to be obtained by

26 $$m^{T} \phi ({\mathbf{x}}) = (\frac{1}{{(\sigma^{2} )^{*} }}{\mathbf{\Sigma \Phi }}^{T} {\mathbf{t}})\phi ({\mathbf{x}})$$

and variance by:

27 $$\sigma^{2} ({\mathbf{x}}) = \left( {\frac{1}{{(\sigma^{2} )^{*} }}} \right)^{ - 1} + \phi ({\mathbf{x}})^{T} {\varvec{\Sigma}}\phi ({\mathbf{x}})$$

where $\phi ()$ is vector of basis functions with non-zero elements for relevance vectors and zeros for the rest.

## Medium-term-load-forecasting using kernel machine regression

**Fig. 1:**
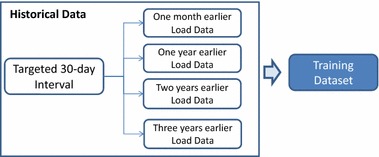
Composition of training datasets used for MTLF

Electricity load demand is a highly volatile signal and depends upon various factors such as: climate, day of the week, season. Capturing the dynamics of all those factors requires the use of appropriate datasets for training the kernel machines. In the present work the training datasets are consisted of historical electric load data *of 1* *month, 1* *year, 2* *years* and *3* *years* earlier than the “target 30-day interval”. For convenience, Fig. 1 depicts the way training datasets are composed.

In the current manuscript we focus on applying kernel machine regression for medium term load forecasting. In particular, we adopt two kernel based methods for MTLF:

(i) Gaussian process regression model equipped with a Gaussian kernel, and
(ii) Relevance vector regression model equipped with a Gaussian kernel.

It should be noted that both forecasting models are kernel machines that are modeled using the Gaussian kernel whose analytical formula is given below (Bishop 2006):

28 $$k(x_{1} ,x_{2} ) = \exp \left( { - \left\| {x_{1} - x_{2} } \right\|^{2} /2\sigma^{2} } \right)$$

with *σ*^2^ denoting a kernel parameter evaluated using the training data.

**Fig. 2:**
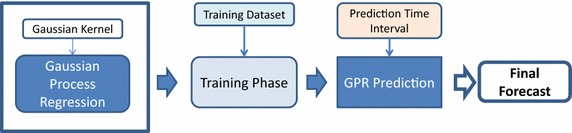
Forecasting process using Gaussian process regression

**Fig. 3:**
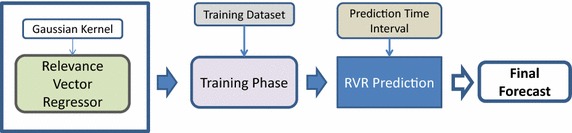
Forecasting process using relevance vector regression

The block diagrams of applying GPR and RVR models in MTLF are presented in Figs. 2 and 3 respectively. We observe the process of forecasting being the same for both kernel machines; the difference lies in the model, i.e., GPR against RVR. Initially, the kernel machine is trained using the training data aiming at evaluating its kernel parameters. Once training ends, the model is suitable for prediction making. To that end, the trained kernel machine provides the final forecasts on the electricity load demand with respect to a predetermined ahead-of-time horizon. The above process is repeated for both kernel machines in every targeted time interval.

In our study, we aim at making daily predictions for a 30-day-ahead horizon. Thus, the goal is to predict the load demand for every day in the next 30 days (overall 30 predicted values). To that end, we have our forecasters making predictions on a monthly basis (January–December) and therefore our study falls within the purpose of MTLF.

## MTLF results

### Problem statement

We apply the presented forecasters to medium term load forecasting for electricity demand load data obtained from the New England ISO (last accessed in 2015) for the period January 2004–August 2011. In particular, we analyze historical load datasets that represent the daily load demand in one of the hubs of the New England ISO Area. Taking into consideration the historical data at our disposal, the forecasters are applied to forecasting demand from January 2007 to August 2011.

**Fig. 4:**
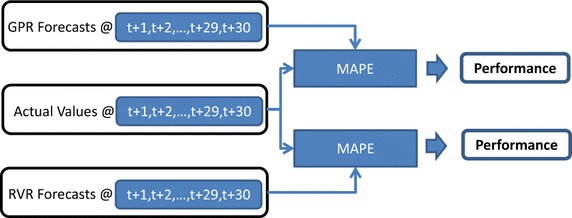
Process of computing MAPE regarding kernel machine forecasters

The kernel machine regression models have been applied to medium term load forecasting; the results are recorded and compared with respect to mean average percentage error (MAPE):

29 $$MAPE = \frac{100}{N}\sum\limits_{t = 1}^{N} {\left| {\frac{{R_{t} - P_{t} }}{{R_{t} }}} \right|}$$

with *R*_*t*_, *P*_*t*_ being the real and predicted value at step *t*, and *N* is the number of timepoints considered in the prediction interval. In the current work, we obtain *N* = 30 as also indicated in Fig. 4, where the forecasting assessment procedure is depicted. Furthermore, the obtained results are compared to those taken with the statistical model of the autoregressive moving average(2,2), i.e., ARMA(2,2) (Huang and Shih 2003) with the ARMA coefficients to be determined by the Alkaline Information Criterion (AIC) (Alamaniotis et al. 2012).

### Test results

**Fig. 5:**
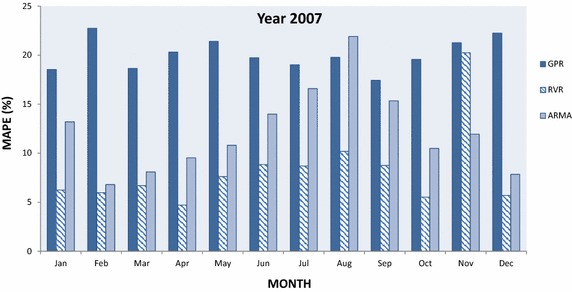
Average per month MAPE results obtained by kernel machine forecasters, i.e., GPR and RVR, as well by ARMA(2,2) for year 2007

**Fig. 6:**
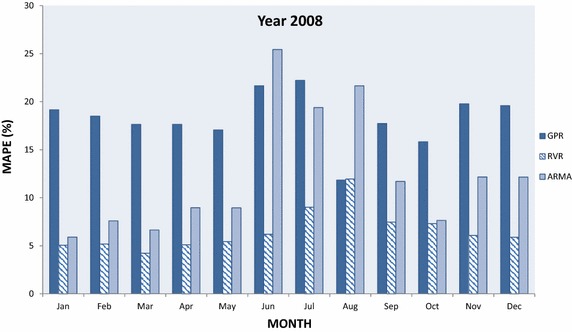
Average per month MAPE results obtained by kernel machine forecasters, i.e., GPR and RVR, as well by ARMA(2,2) for year 2008

**Fig. 7:**
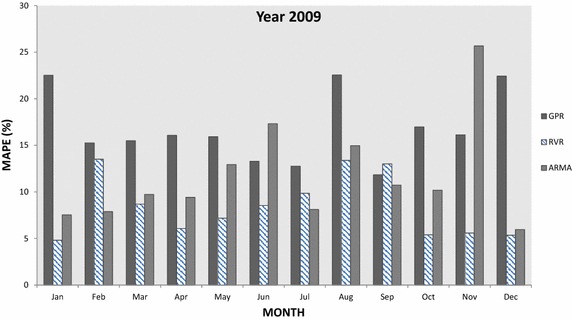
Average per month MAPE results obtained by kernel machine forecasters, i.e., GPR and RVR, as well by ARMA(2,2) for year 2009

**Fig. 8:**
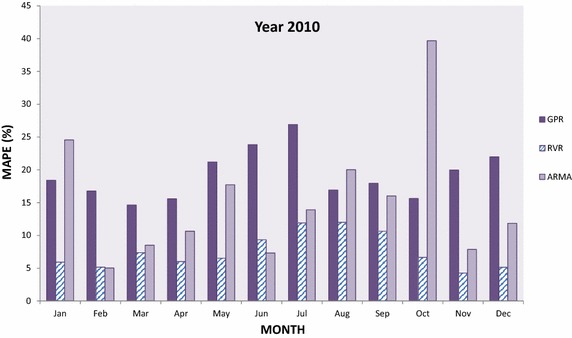
Average per month MAPE results obtained by kernel machine forecasters, i.e., GPR and RVR, as well by ARMA(2,2) for year 2010

**Fig. 9:**
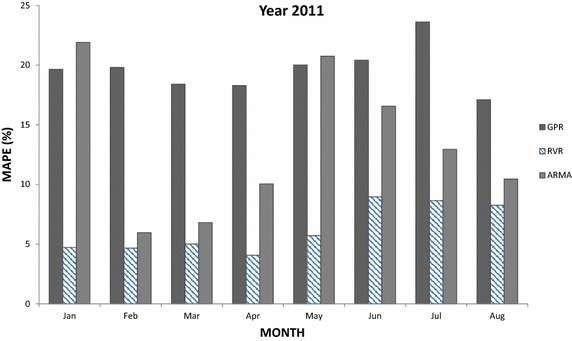
Average per month MAPE results obtained by kernel machine forecasters, i.e., GPR and RVR, as well by ARMA(2,2) for year 2011

In this section, GPR and RVR have been applied in medium-term load forecasting and the results obtained for the designated time interval are plotted and compared to each other as well to results obtained with ARMA(2,2). In particular Figs. 5, 6, 7, 8 and 9 present the computed MAPE during the tested (almost) 5 year period for GPR, RVR and ARMA(2,2) forecasters. Results are depicted in terms of monthly intervals, giving 12 results for years 2007–2010 and 8 results for year 2011 (it was mentioned above that available tested data are from January 2007 to August 2011).

Figure 5 exhibits that RVR forecaster provides more accurate daily predictions for a month-ahead-horizon (i.e., 30-day ahead horizon) with respect to MAPE. In particular RVR gives the best performance for all months but November, where ARMA is the best forecaster. GPR gives the worst performance for all months in 2007 except for August. In 2008 data, Fig. 6 exhibits RVR as the best performing forecaster in all tested months except for August, where it is slightly outperformed by GPR. ARMA(2,2) performance is better than GPR and worse than RVR in the majority of the cases, with the exception of June and August 2008; for the latter months the ARMA forecasts are the least accurate among all forecasters.

In Fig. 7, we observe that RVR once more provides the best performance in the majority of the cases for year 2009—with the exception of February, July and September. For the same time interval (i.e., 2009), GPR provides the worst performance among three forecasters with a few exceptions. Furthermore, results for year 2010 presented in Fig. 8 drive to similar conclusions as earlier: RVR is the best forecaster in the majority of the cases (in 10 out of 12), GPR the worst in most of them, while ARMA is the worst in two cases (January and October) and the best in other two (February and June). Additionally, in Fig. 9 provides the MAPE results for the first 8 months of year 2011: RVR clearly outperforms the other two forecasters in all cases, GPR provides the least accurate predictions in February, March, April, June, July and August, and ARMA is the least accurate for January and May.

**Fig. 10:**
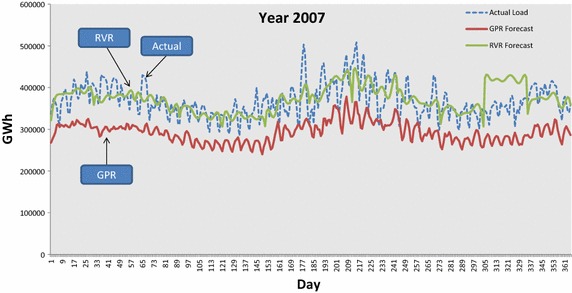
GPR and RVR predicted values against actual load demand for year 2007

**Fig. 11:**
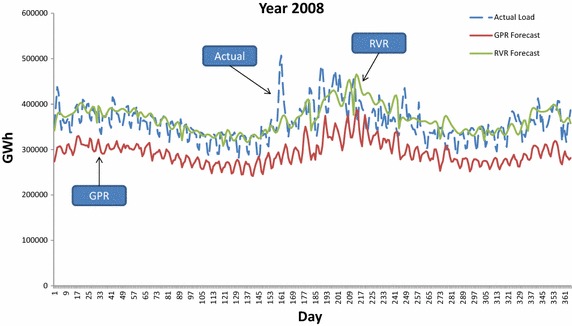
GPR and RVR predicted values against actual load demand for year 2008

In addition to monthly results, we present in Table 1 a yearly summary of the MAPE results obtained by each of the three forecasters. In particular, the average MAPE per tested year with respect to GPR, RVR and ARMA are given in the columns of Table 1. Yearly averages exhibit that RVR is by far the most accurate forecaster for all tested years. The second most accurate is the ARMA model, with the GPR kernel machine to be the least accurate. For demonstration purposes, the forecasted demand by GPR and RVR are plotted against the actual demand for years 2007 and 2008 in Figs. 10 and 11 respectively. In both Figures we clearly observe that the RVR forecaster follows the actual demand closer than GPR.

**Table 1:** Average per year MAPE obtained by GPR, RVR and ARMA forecasters

Forecaster	MAPE (%)				
	Year 2007	Year 2008	Year 2009	Year 2010	Year 2011
GPR	20.0549	18.2197	16.7651	19.1449	19.6651
RVR	*8.2596*	*6.5793*	*8.4424*	*7.5811*	*6.2573*
ARMA(2,2)	12.2093	12.3496	11.6999	15.2576	13.1817

The lowest values are italicized

**Fig. 12:**
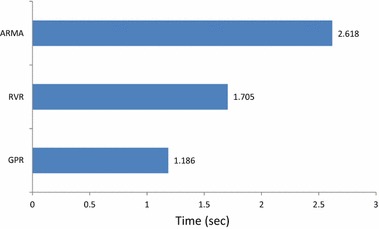
Average execution times obtained by kernel machine forecasters, i.e., GPR and RVR, as well by ARMA(2,2)

In addition to MAPE criterion, we have also computed the squared correlation coefficient (*R*^2^) between the predicted and the actual load values, despite the fact that *R*^2^ is not very common criterion in load forecasting. This criterion does not express directly the performance of the forecaster but it shows how good a forecaster might be constructed from the predicted values. The obtained average per year *R*^2^ for each of the three forecasters is given in Table 2 where we observe that the ARMA provides the highest value for years 2007, 2008 and 2010, while RVR for 2009 and 2011. Overall combining observations from MAPE and *R*^2^ from Tables 1 and 2, we may conclude that the ARMA captures the general trend of the load signal adequately in more cases than RVR but it is less accurate than RVR. In addition, it is slower in execution than both the kernel machines, with the GP to be the fastest. Figure 12 shows the average execution time of the models tested in this paper; models were run on an Intel i5 core laptop computer.

**Table 2:** Average per year squared correlation coefficient (R^2^) obtained by GPR, RVR and ARMA forecasters

Forecaster	Squared correlation coefficient (R^2^)				
	Year 2007	Year 2008	Year 2009	Year 2010	Year 2011
GPR	0.282	0.227	0.201	0.255	0.381
RVR	0.192	0.374	*0.286*	0.295	*0.438*
ARMA(2,2)	*0.444*	*0.533*	0.185	*0.320*	0.120

The best coefficients are italicized

Therefore, we observe that depending on the selected model kernel machine may provide high accurate MLTF, as taken by RVR, or may provide low accuracy, as is the case with GPR.

## Conclusion

The application of two types of kernel machines for medium-term load forecasting has been presented in this paper. The kernel machines studied are GPR and RVR whose performance is tested on actual historic data collected at the New England Area on a daily basis up to a month, with the tested time period being from January 2007 to August 2011. In addition, both forecasters are also compared to the ARMA(2,2) statistical tool that has been widely used in time series forecasting.

Obtained results show the superiority of RVR over the other two tested methods with respect to MAPE and *R*^2^. On a monthly comparison RVR provided the best accuracy in the majority of the cases while it is by far the best forecaster on a yearly based comparison. However, it should be emphasized that the kernel machines are equipped with a Gaussian kernel, which is the only kernel being tested in the current work; testing of other kernel functions is left for future work.

In addition, the promising method of core vector regression (Li and Liu 2010) will also be examined either as an independent forecaster or in combination with RVR and GP. Combination of kernel machines exhibits high potency for providing highly accurate medium term load predictions.
